# Rare adrenal cavernous hemangioma: a case report highlighting diagnostic challenges

**DOI:** 10.3389/fsurg.2023.1293925

**Published:** 2023-11-10

**Authors:** Ryan Michael Antar, Christian Mark Farag, Kirolos Youssef, Vincent Xu, Arthur Drouaud, Noah Panitch, Zoon Tariq, Ali Alzeer, Michael J. Whalen

**Affiliations:** ^1^ Department of Urology, George Washington University School of Medicine, Washington, DC, United States; ^2^ Department of Medicine, George Washington University School of Medicine, Washington, DC, United States; ^3^ Department of Pathology, George Washington University School of Medicine, Washington, DC, United States

**Keywords:** adrenal mass, adrenalectomy, cavernous hemangioma, adrenaloma, incidentaloma, hemangioma

## Abstract

**Introduction:**

Adrenal cavernous hemangiomas are rare benign vascular tumors that pose significant diagnostic challenges. Despite their benign nature, features overlapping with malignancies often complicate management decisions.

**Case presentation:**

A 64-year-old male presented with a 4.4 cm necrotic left adrenal mass discovered incidentally on imaging. His medical history included papillary thyroid carcinoma, with subsequent thyroidectomy and radioactive iodine ablation. Evaluations for hiccups revealed multiple lung nodules, hypertrophic cardiomyopathy, and anemia. Given the patient’s previous cancer history, elevated aldosterone/renin ratio, and mass size, our multidisciplinary tumor board decided to proceed with a left adrenalectomy. Post-surgical pathology confirmed a diagnosis of adrenal cavernous hemangioma.

**Conclusion:**

The occurrence of ambiguous adrenal mass with other pathologies, such as our patient’s papillary thyroid carcinoma, complicates the diagnostic and therapeutic landscape. As demonstrated in our case, opting for surgery remains a viable solution for adrenal cavernous hemangiomas, especially for masses greater than 4 cm. Interdisciplinary collaboration, exemplified by our tumor board’s decision-making process, is crucial for optimal management. This case underscores the need for a multifaceted approach when confronting adrenal masses with such diagnostic ambiguity.

## Introduction

Adrenal cavernous hemangiomas are rare benign vascular tumors that present significant diagnostic challenges in urologic practice. Since first being recognized in the mid-1950s, adrenal cavernous hemangiomas have only been documented in approximately 70 case reports (1). Often identified during incidental imaging, adrenal cavernous hemangiomas compound the complexity of the diagnostic workup associated with adrenal incidentalomas (2). Despite the increasing prevalence of adrenal incidentalomas, driven primarily by the growing use of cross-sectional abdominal imaging, there remains ambiguity regarding their management guidelines (3). The decision to proceed with adrenalectomy, the recommended treatment for adrenal hemangiomas, often requires intricate collaboration spanning multiple specialties, encompassing imaging and biochemical testing. Here, we introduce a case of adrenal cavernous hemangioma diagnosed through surgical pathology and complicated by a history of papillary thyroid carcinoma, anemia, and abnormal hormonal testing results. Additionally, we present a thorough review of the literature and discuss management strategies and the inherent diagnostic challenges tied to this specific adrenal incidentaloma subtype.

## Case presentation

### Clinical course

We report the case of a 64-year-old male who presented with a 4.4 cm necrotic left adrenal mass found incidentally on imaging. The patient has a history of papillary thyroid carcinoma and underwent partial thyroidectomy in May 2010, followed by complete thyroidectomy in July 2010 and radioactive iodine ablation in September 2010. Our patient has been on suppressive and substitutive treatment with thyroid hormone since his surgery in 2010. However, increased treatment doses were required in 2020 due to challenges in maintaining optimal thyroid hormone levels.

**Figure 1:**
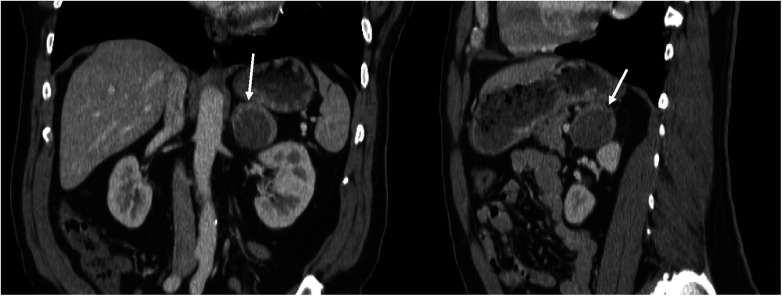
Coronal and sagittal views of CT abdomen with contrast, showing 4.4 cm left adrenal mass with peripheral enhancement and central heterogeneous hypoenhancement, July 2022.

In February 2022, the patient underwent a diaphragm fluoroscopy (fluoroscopic Sniff test) during a workup for intractable hiccups by his endocrinologist, which revealed multiple lung nodules. A subsequent computed tomography (CT) scan of the thorax in August 2022 revealed suspicious solid lung nodules, with the largest being 1.5 cm, along with a 4.4 cm left adrenal mass with peripheral enhancement and central heterogeneous hypoenhancement suggestive of necrosis. The differential diagnosis provided by the attending radiologists at the time of imaging included adrenal myelolipoma, metastatic disease from the patient’s previous thyroid cancer, or primary adrenocortical carcinoma. However, there was no discernable macroscopic fat within the mass, making the diagnosis of myelolipoma less likely. The adrenal mass was better visualized on CT Abdomen from the month prior (Figure 1). A routine thyroid ultrasound to evaluate for potential recurrence, also in August 2022, demonstrated a benign 0.6 cm thyroid bed nodule classified as TI-RADS 2. As part of his workup for both the adrenal mass and lung nodules, he underwent a positron emission tomography CT (PET-CT), revealing a mildly hypermetabolic left adrenal mass but no activity in the thyroid region. Subsequently, a radionuclide iodine scan to assess for metastatic thyroid cancer was performed, which was negative. A CT-guided lung nodule biopsy was attempted to characterize these masses further but could not be completed due to proximity to the patient’s rib. As such, a bronchoscopy-guided biopsy was planned in November 2022. However, preoperatively, the patient experienced a syncopal episode and was found to have hypertrophic cardiomyopathy. The patient thus had an implantable cardioverter defibrillator (ICD) placed in January 2023 and underwent a lung nodule biopsy in March 2023, which was negative for malignant cells but considered largely non-diagnostic.

Our patient’s blood pressure was well-controlled by anti-hypertensive medications (beta blocker & calcium-channel blocker) and did not display any stigmata of Cushing’s Syndrome or pheochromocytoma, later confirmed by biochemical testing. Notably, the patient was also anemic during his clinical course, found to be 7.5 gm/dl in November of 2022. Our patient’s anemia slightly improved to 8.5 gm/dl one week before his operation. Before surgery, his laboratory studies were notable for a high aldosterone/renin ratio of >117.4 ng/dl per ng/ml/hr (reference range 0.0–30.0), with aldosterone at 19.6 ng/dl (reference range 0.0–30.0) and low renin at <0.167 ng/ml/hr (reference range 0.167–5.380) in April 2023. Plasma metanephrines collected simultaneously were within normal limits (normetanephrine 98.3 pg/ml, reference range 0.0–285.2; metanephrines 35.3 pg/ml, reference range 0.0–88.0), lessening the suspicion for pheochromocytoma.

The differential diagnosis remained broad, with options between metastatic thyroid carcinoma, adrenal cortical carcinoma, and functional adenoma. Our institution’s multidisciplinary tumor board decided to proceed with a left robotic adrenalectomy and left retroperitoneal lymph node dissection in June 2023. Intraoperatively, no visceral injury was noted, and there was no evidence of metastatic disease. Moreover, there was no suspicious renal hilar or para-aortic lymphadenopathy. A branching renal vein and a single renal artery were noted. The left gonadal artery was spared, and the left adrenal gland was removed with the mass intact without capsule rupture and injury to the adjacent kidney, splenic hilum, or pancreas. Postoperatively, the patient was discharged after one day.

### Pathology

**Figure 2:**
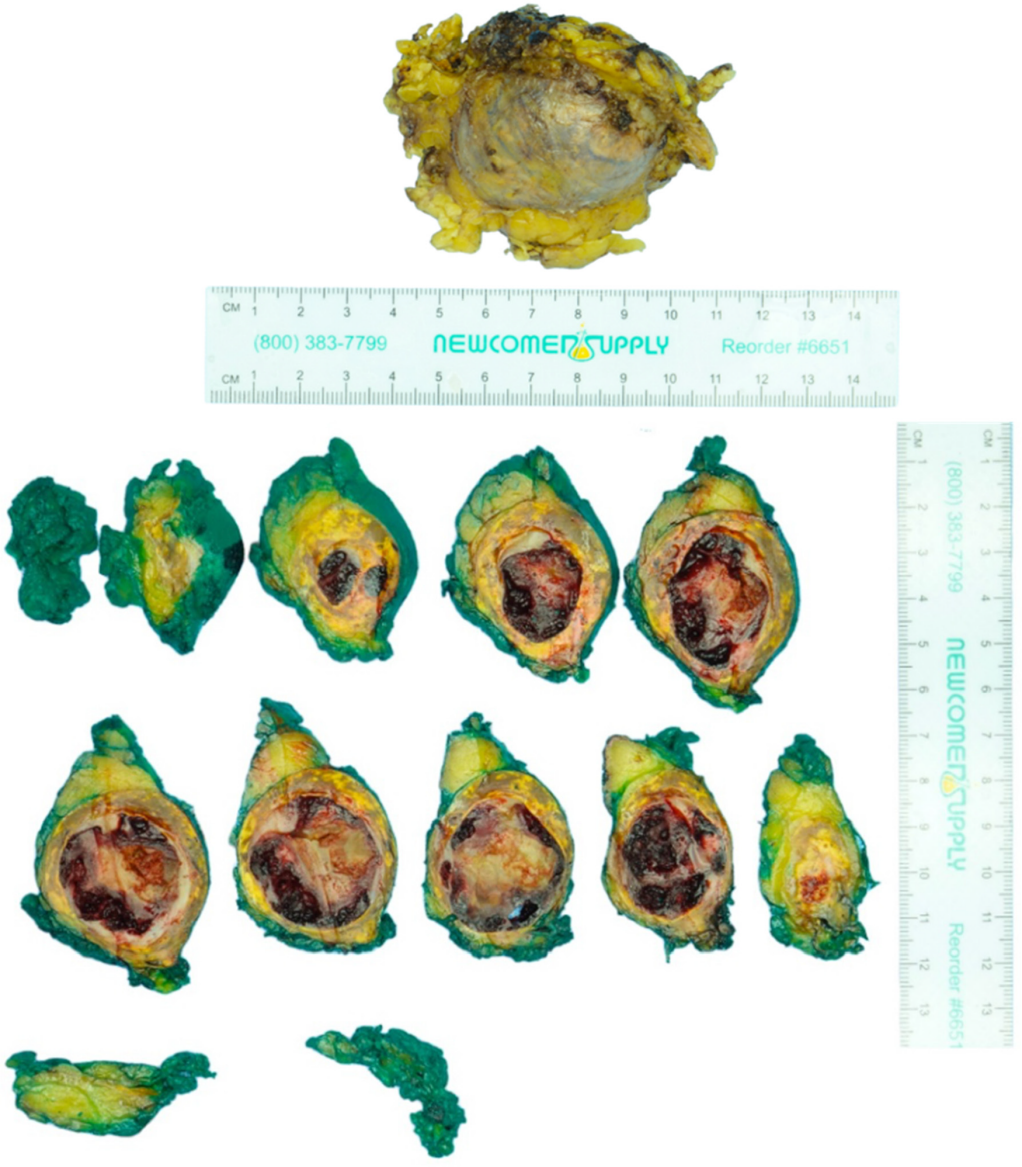
Pathology findings of left adrenal mass: macroscopic. The specimen consists of a yellow-tan unoriented left adrenalectomy weighing 55 g and measuring 7.5 × 6.5 × 3.7 cm. The specimen is inked green and serially sectioned to reveal a tan to yellow-orange circumscribed hemorrhagic 7.0 × 4.1 × 3.6 cm mass.

**Figure 3:**
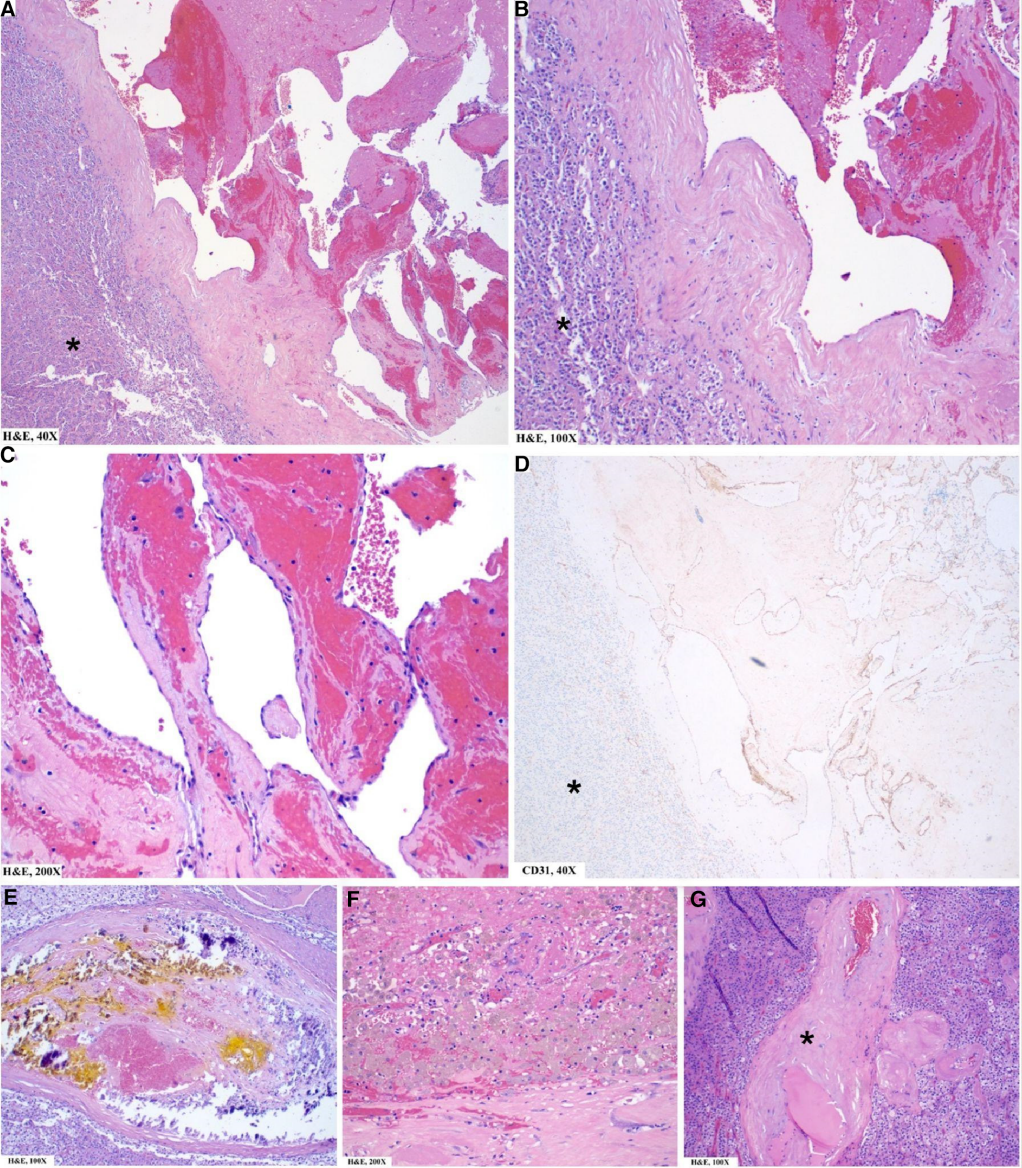
Pathology findings of left adrenal mass: microscopic. (**A**–**C**) Well-circumscribed vascular lesion (note demarcation from normal adrenal*) consisting of variously sized dilated thin-walled vascular channels lined by a single layer of flattened endothelial cells. (**D**) CD31/PECAM1 (a highly sensitive and specific marker of vascular differentiation) is positive in the lesional cells (but negative in normal adrenal* except for normal vasculature within the adrenal). (**E**,**F**) Focal areas of calcification (**E**) and thrombosis with hemosiderin-laden macrophages (**F**) are noted and are chronic features in the development of this lesion. (**G**) Few areas of eosinophilic amorphous material (*) around vessels are identified concerning for amyloid deposition. Not shown here is the Congo Red special stain, which was performed with adequate controls and negative.

The surgical specimen submitted as a left adrenal mass (Figure 2) was examined microscopically to reveal a blood-filled vascular proliferation of thin-walled vessels with intervening stroma with focal thrombosis, consistent with a cavernous hemangioma (Figure 3). Thrombosis and calcifications most likely represent features of chronicity (Figures 3E,F). Immunohistochemical stains were performed with adequate controls and showed CD31 (vascular marker) to be positive in the lesional cells and Ki-67 (proliferation marker) as low. Congo red special stain was also performed to rule out amyloid deposition (Figure 3G).

The histopathological differential diagnoses include other morphologic variants of hemangioma, a lymphangioma (ruled out based on the presence of red blood cells in vessels; lymphangiomas usually show empty vessels), angiosarcoma (ruled out by the absence of cytologic atypia, mitotic figures, necrosis, and a low proliferation index).

## Discussion

Adrenal masses are relatively common, with a 5%–10% prevalence depending on the age and population studied (4). However, adrenal hemangiomas are rare, and their pathogenesis has yet to be fully understood. Congenital or hereditary factors are usually involved in skin, brain, and liver hemangiomas. These benign tumors present diagnostic challenges as their non-specific imaging characteristics can encompass benign and malignant entities, mainly mimicking adrenocortical carcinomas (5). Specifically, heterogenous features such as calcifications, necrosis, and hemorrhage raise concern for adrenocortical carcinomas when assessing adrenal masses. This was the case for our patient, whose CT scan revealed peripheral calcifications and central necrosis. Chua et al. compiled all known hemangiomas between 2019 and 2021, adding to an existing database of adrenal hemangiomas by Degheili et al. characterizing adrenal hemangiomas (6). Most adrenal hemangiomas tend to be found incidentally, and patients tend to be asymptomatic at discovery, similar to our patient. However, patients who report clinical symptoms tend to demonstrate vague abdominal discomfort and pressure in the lower abdominal quadrants, radiating to the flanks (7). These clinical manifestations tend to be secondary to the mass effect from the size of the adrenal tumor. The sizes of such tumors in the literature have varied significantly, ranging from 2.5 to 25 cm in diameter (8, 9). Interestingly, of 73 characterized hemangiomas, 61% occurred in females, although our case occurred in a male. Our patient also had a left-sided hemangioma, although these tumors tend to be seen equally between the left and right. Additionally, 70% of patients in one case series had a normal metabolic workup. Our patient presented with an elevated aldosterone/renin ratio, but his aldosterone levels were within normal limits while renin secretion was suppressed, indicating aldosterone release independent of the renin-angiotensin-aldosterone system (RAAS) pathway.

The association between adrenal masses and other pathologies, as seen in our patient’s papillary thyroid carcinoma history and hypertrophic cardiomyopathy, may add complexities to diagnostic and therapeutic decisions. Although rare, four published cases have described the metastasis of papillary thyroid cancer to the adrenal glands (10). Thus, the plausibility of metastasis to the adrenal glands certainly factored into our differential diagnosis. The patient under our care exhibited negligible clinical symptoms, devoid of any signs of abdominal discomfort or pressure. Given our patient’s original complaint of hiccups, it is worth recognizing the link between cavernous hemangiomas of the central nervous system and intractable hiccups (11). Following a neurology consultation, our patient’s hiccups are most likely not neurological in origin but likely stemming from his pulmonary nodules irritating his diaphragm. Nevertheless, given the history of cancer to the thyroid, it was deemed appropriate to adhere to the prescribed protocols and carry out the adrenalectomy.

Preoperatively, our patient’s hemoglobin was 8.5 gm/dl, indicating anemia during his care period. There have been two documented cases aside from our patient who presented with anemia alongside abdominal symptoms, potentially due to the sequestration of the blood in conglomerate vessels to the hemangioma (7). A thorough comprehension of the molecular pathophysiology of hemangiomas as they relate to the red blood cell profile is imperative, as anemia may arise from anemia of chronic disease (ACD) or the angiogenesis associated with the hemangioma itself. However, given the benign nature of the hemangioma in our patient, it is reasonable to infer that ACD likely does not contribute to the observed anemic condition, as ACD is primarily associated with malignancies (12). The complete etiology of hemangiomas remains elusive; nonetheless, existing literature substantially supports the connection between oncogenic modifications in hemangiomas and the stimulation of tumor angiogenesis. Evidence indicates that somatic mutations resulting from loss of heterozygosity on chromosome 5q could initiate the formation of hemangiomas (13, 14). Interestingly, another case report in the literature also describes a patient with a 10-year history of thyroidectomy and the subsequent discovery of a non-functional left adrenal cavernous hemangioma, similar to our patient (15). Post-thyroidectomy, a compensatory increase in thyroid-stimulating hormone (TSH) levels may be observed, which may theoretically influence angiogenic pathways, such as vascular endothelial growth factor (VEGF) upregulation (16). Increased VEGF activity has been implicated in the pathophysiology of hemangiomas (17). Thus, it becomes a plausible hypothesis that the angiogenic processes within the hemangioma sequester red blood cells for their proliferation, consequently contributing to the concurrent anemia of our patient. Furthermore, a noteworthy consideration is the potential impact of hemangioma size on the resulting anemia. One case report described a 79-year-old male with a hemangioma measuring 20.8 cm, concurrent with a hemoglobin level of 7.0 gm/dl (7). In contrast, our patient’s preoperative hemoglobin of 8.5 gm/dl with his 4.4 cm hemangioma presenting on the lower end of the spectrum for such formations. Though these associations are speculative, they are compelling and warrant further investigation to enhance our understanding of the factors linked to hemangioma formation, especially in individuals with a history of cancer, such as our patient. Drawing parallels between individual cases of adrenal hemangiomas provides insights into tailoring patient care. In the same vein, collaborative forums, such as a tumor board in our case, exemplify how an interdisciplinary approach to understanding and managing these conditions optimizes cancer care.

Tumor boards are associated with changes in patient management and improved clinical outcomes, potentially attributable to clinicians’ opportunity to exchange knowledge to explore alternative treatment modalities while adhering to clinical guidelines (18). Coordinated care through tumor board discussions and multi-disciplinary teams may also improve patient satisfaction (19). A recent study by the Michigan Urological Surgery Improvement Collaborative utilized a virtual tumor board for 50 complex renal masses and found that 93% of urologists had increased confidence in their case management after discussions (20). Additionally, the virtual tumor board confirmed the physician’s initial approach in 36% of cases and offered alternative approaches in 16%, emphasizing the crucial role that multi-institutional and multi-disciplinary discussions have in refining treatment strategies for individual patients. Such virtual platforms can overcome traditional barriers like time constraints, allowing for a more comprehensive debate on complicated cases to improve care quality.

Tumor boards can be beneficial in discussing workup for adrenal hemangiomas due to the breadth of the differential diagnosis. Biopsy presents unique challenges. Given the nature of these vascular tumors, biopsy carries the risk of causing adrenal hemorrhage. Additionally, some of these tumors are metabolically active in the case of pheochromocytomas and may be associated with a catecholamine surge with biopsy and must be pretreated with alpha-blockers. Moreover, while hemangiomas are benign tumors, the differential diagnosis includes several malignant tumors, which leaves observation as a potentially harmful option to the patient. Our case was complicated by radiographic findings, which included peripheral enhancement and central heterogeneous hypoenhancement suggestive of necrosis, suggesting that there was a possibility that this tumor was malignant. Despite these complexities, in cases where adrenal masses are >4 cm in size, such as the one presented in this case, adrenalectomy often emerges as the preferred solution (5). One option we considered was performing adrenal venous sampling to lateralize the source of aldosterone before proceeding with the adrenalectomy. However, the radiographic appearance (mass >4 cm and necrosis) strongly suggested malignancy. As such, given the necessity for surgical intervention, we chose not to pursue adrenal venous sampling. Malignancy and predisposition to bleeding advance the management of the mass to surgical resection. A more conservative approach may be appropriate when managing adrenal masses smaller than 4 cm. This decision should factor in patient characteristics such as age, overall health, and history of malignancies. Additionally, the features of the tumor and the potential benefits of surgical intervention must be considered. Longitudinal observation is preferable, especially since adrenalectomy is reserved exclusively for malignant masses and can help alleviate pressure accumulation (7). As such, a decision was made to remove the entire adrenal gland without an adrenal biopsy. Multidisciplinary approaches allow full consideration of all known information, offering the best course of action for each unique patient case.

## Conclusion

Adrenal cavernous hemangiomas are rare benign tumors that pose significant clinical and radiologic diagnostic challenges due to overlapping findings with malignancies. Our case report underscores the nuances in managing such adrenal masses, particularly when they coexist with other confounding conditions. As demonstrated through our tumor board’s decision for adrenalectomy, the role of interdisciplinary collaboration emphasizes the importance of a multifaceted approach in managing these complex cases.

## Data Availability

The original contributions presented in the study are included in the article/Supplementary Material, further inquiries can be directed to the corresponding author.
